# The “chapeau de gendarme” sign in focal epilepsy: A systematic review

**DOI:** 10.1002/epd2.70048

**Published:** 2025-05-27

**Authors:** Katharina Moser, Dorottya Cserpan, Antonio Giulio Gennari, Andrea Rüegger, Francine Chassoux, Georgia Ramantani

**Affiliations:** ^1^ Department of Neuropediatrics University Children's Hospital, University of Zurich Zurich Switzerland; ^2^ Swiss Children's Rehab University Children's Hospital Zurich, University of Zurich Affoltern am Albis Switzerland; ^3^ MR‐Research Centre University Children's Hospital Zurich Zurich Switzerland; ^4^ Department of Neurosurgery Lariboisière Hospital Paris France; ^5^ Children's Research Center University Children's Hospital Zurich, University of Zurich Zurich Switzerland

**Keywords:** anatomo‐clinical correlation, chapeau de gendarme, epilepsy surgery, focal epilepsy, ictal pouting, ictal semiology, systematic review

## Abstract

The “chapeau de gendarme” sign, also known as “ictal pouting,” is a distinctive facial expression observed in focal epilepsy, characterized by a turned‐down mouth with symmetrical lip and chin contraction. This systematic review investigates its clinical relevance, anatomical origins, and diagnostic value, particularly in presurgical evaluation. The sign is most commonly associated with mesial frontal epilepsy, particularly involving the anterior cingulate cortex (ACC), though moderate evidence links it to a broader network including insulo‐opercular and temporo‐parietal regions. Variability in semiologic descriptions and the absence of standardized definitions present challenges to its consistent recognition. Focal cortical dysplasia (FCD) was identified as the most frequent pathological substrate linked to this sign, particularly in MRI‐positive cases, while nearly half of the reviewed cases were MRI‐negative emphasizing the importance of localizing signs such as the “chapeau de gendarme” in cases of suspected FCD. FDG‐PET consistently showed hypometabolism in relevant regions, supporting its utility in identifying the epileptogenic zone in MRI‐negative cases. The propagation of ictal discharges across interconnected brain regions highlights the involvement of specific network dynamics in generating this semiology. The “chapeau de gendarme” sign holds significant value in presurgical evaluations, particularly as part of a multimodal assessment integrating neuroimaging and electrophysiological data. Despite the descriptive nature of included studies and limited photo documentation, the sign remains a valuable diagnostic tool. Further research is needed to standardize reporting and clarify its role in guiding surgical management, especially in MRI‐negative focal epilepsy.

**Key points**

- The chapeau de gendarme sign (ictal pouting) is a distinctive ictal facial expression that may aid in localizing seizure onset in focal epilepsy.
- It is most commonly associated with mesial frontal epilepsy, particularly involving the anterior cingulate cortex, but may also reflect early propagation within a broader network.
- Focal cortical dysplasia, especially type II, is the most frequent histopathological substrate.
- Recognition of this semiology, particularly in MRI‐negative cases, can guide presurgical evaluation and inform SEEG planning.

## 1 INTRODUCTION

The “chapeau de gendarme” sign, also known as “ictal pouting,” is a distinctive semiologic feature observed in focal epilepsy. This facial expression, characterized by a turned‐down mouth or inverted smile with symmetrical contraction of the lips and chin, resembles the hats worn by gendarmes during the French Revolution.^1^ Initially described by French epileptologists and introduced to English literature only a decade ago,^1^ the “chapeau de gendarme” sign is recognized as a potential localizing feature, most often associated with focal cortical dysplasia (FCD),^1^, ^2^, ^3^, ^4^ one of the most common substrate of focal epilepsy amenable to surgery, especially in children. First reported as rare, it has since been increasingly identified worldwide and gained attention in clinical practice.

Depending on the medical and cultural context, this sign is also described by various terms that do not always refer to the same feature, including ictal pouting,^1^, ^3^, ^5^, ^6^, ^7^, ^8^, ^9^ grimacing,^10^ tonic facial contraction,^11^ and “Kabuki visage”.^12^ This variation complicates its identification and classification. The term “pouting” was adopted to translate the French expression “chapeau de gendarme,” which is untranslatable in English but does not fully capture the same expression. This semantic barrier likely contributes to differing interpretations and reports. In addition, detection of this sign can be challenging, as it remains a lesser‐known feature, primarily linked to frontal lobe epilepsies, where semiologic features may be overlooked during sleep‐related seizures, particularly when the patient is lying prone with their head on a pillow. Although changes in facial expression are not uncommon in frontal epilepsy, they are often part of a broader set of complex behaviors, such as sudden agitation or bizarre actions, and are rarely described as isolated features. However, despite some debate, the “chapeau de gendarme” sign is recognized as reflecting a negative emotional expression rather than a primary motor sign.^1^, ^2^

Stereoelectroencephalography (SEEG) studies^1^, ^2^, ^11^, ^13^ have linked this semiology to a frontal lobe network involving the anterior cingulate cortex (ACC), superior frontal gyrus, frontal operculum, and adjacent insular regions, suggesting the involvement of emotional and motor pathways.^1^, ^2^ However, some cases have indicated temporal lobe involvement,^3^, ^7^ raising questions about the exact origin of this facial expression and the potential contributions of other brain regions, either at seizure onset or through propagation.

To address these uncertainties regarding the definition and localizing value of the “chapeau de gendarme” sign, we conducted a systematic review. Our aim is to clarify its neuroanatomical origins and consolidate current knowledge on its role in presurgical evaluation.

## 2 METHODS

### 2.1 Search strategy and eligibility criteria

This systematic review was registered in PROSPERO (International Prospective Register of Systematic Reviews; CRD42024519156) on March 21, 2024. This review was conducted and reported according to the Preferred Reporting Items for Systematic Review and Meta‐Analysis (PRISMA) guidelines.^14^ We systematically searched PubMed, EMBASE, and Scopus, using a broad strategy to capture all descriptions related to “chapeau de gendarme” semiology (e.g., “pouting” or “downward turning of the mouth”^11^; Tables S1–S3). The search was last conducted on December 1, 2024.

We included original, peer‐reviewed research articles without language or time restrictions. Duplicates were removed using Covidence software (Veritas Health Innovation, Melbourne, Australia) and were manually checked. Two independent reviewers (K.M., G.R.) initially screened a random subsample of 200 titles and abstracts with high agreement; one reviewer (K.M.) then screened the remainder. Full‐text eligibility was assessed independently by both reviewers using a predefined scoring sheet to ensure systematic and consistent evaluation of the included studies (Table S4). Case reports were eligible if they described spontaneous ictal signs and symptoms.

Articles were eligible if they provided detailed information on at least one of the following: (1) invasive EEG recordings, (2) epilepsy surgery with postsurgical seizure outcomes, or (3) brain imaging data. Due to the scarcity of reports focused on this specific semiology, we included subgroups from studies where “chapeau de gendarme” was discussed within broader objectives. Articles were included only if they provided individual‐level details for each patient with “chapeau de gendarme” semiology. Cases were eligible if they described “chapeau de gendarme,”^4^, ^15^ “pouting,”^6^ or contained descriptions^1^/images^2^, ^3^, ^4^, ^11^, ^16^ resembling “chapeau de gendarme.” Cases labeled as “grimacing”^17^ were included only if detailed seizure semiology was provided; otherwise, they were excluded.

### 2.2 Data extraction

For each selected publication, we extracted the following variables: author, year, country of publication, number of reported patients, proportion of patients with informative data on “chapeau de gendarme” semiology, adult‐to‐pediatric ratio, sex, age at epilepsy onset, age at surgery, and any data relevant to anatomo‐clinical correlations.

### 2.3 Risk of selection and assessment bias

We evaluated the risk of bias for each publication using an adapted QUADAS‐2^18^ assessment, excluding single case reports. To assess selection bias, we examined whether the study enrolled a consecutive or random sample of patients, clearly defined the sampling method, used a case–control design, and avoided inappropriate exclusions such as omitting more complex cases to achieve more favorable outcomes. Based on these factors, we categorized the risk of selection bias as low, high, or unclear.

Assessment bias was evaluated by rating each publication as low, high, or unclear, based on whether the interpretation of semiology was conducted independently of other data, particularly EEG and MRI findings (i.e., without prior knowledge of these results).

### 2.4 Reliability of the reference standard

We assessed confidence in the reported epileptogenic zone (EZ) for individual subjects using a recently developed method,^19^ which categorizes evidence into four levels based on MRI findings, invasive EEG (iEEG) data, and postsurgical seizure outcomes:

- “very high” confidence: Assigned to cases with Engel class IA outcomes after at least 1 year of postsurgical follow‐up.
- “high” confidence: Assigned to cases with either: (1) a well‐delineated focal lesion suspected to be part of the EZ, (2) a well‐delineated EZ based on all available iEEG data, or (3) an Engel class I outcome (but not specifically IA) after at least 1 year of postsurgical follow‐up.
- “moderate” confidence: Assigned to cases with MRI evidence of hippocampal sclerosis or atrophy suspected to be part of the EZ.
- “low” confidence: Assigned to cases with normal MRI or with multilobar, multifocal or poorly delineated lesions, or a poorly delineated EZ based on iEEG data, or Engel class II–IV postsurgical outcome, provided the EZ was fully removed.

Cases in which the EZ was not fully removed and no second surgery was performed were excluded from grading. If multiple confidence levels were indicated, the postsurgical outcome took precedence over iEEG and MRI findings, while iEEG conclusions prevailed over MRI findings. For each selected paper that included at least two patients with “chapeau de gendarme” semiology, we reported the proportion of patients corresponding to each confidence level.

### 2.5 Overall evidence

The overall evidence was evaluated using the GRADE system (Grading of Recommendations Assessment, Development and Evaluation),^20^ which categorizes the level of evidence as follows:

- Very low reliability: The true effect is likely very different from the estimated effect.
- Low reliability: The true effect might be significantly different from the estimated effect.
- Moderate reliability: The true effect is probably close to the estimated effect.
- High reliability: There is high confidence that the true effect is similar to the estimated effect.

### 2.6 Statistical analysis

We performed a descriptive analysis of the patient cohort. Continuous variables are reported as mean, standard deviation (SD), and range, while categorical variables are expressed as percentages. To evaluate differences in occurrences across brain regions, we applied Pearson's chi‐squared test. When significant differences were identified, pairwise proportion tests with Holm correction for multiple comparisons were conducted as post‐hoc analyses to compare distributions between specific brain regions. Furthermore, pairwise ratios of occurrences across brain regions were calculated to provide a relative measure of their reporting frequencies.

## 3 RESULTS

### 3.1 PRISMA flow diagram

**FIGURE 1:**
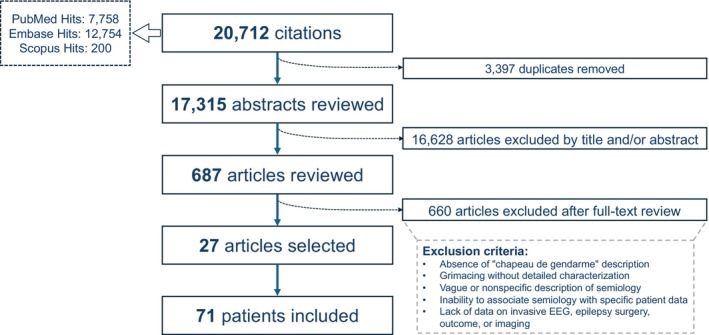
Flow chart of the literature search.

The PRISMA flow diagram (Figure 1) outlines the study selection process. We initially identified 20 712 citations, screened 17 315 abstracts, and reviewed 687 full‐text articles, finally including 27 studies, comprising a cohort of 71 patients, in our analysis. All included studies were published in English.

### 3.2 Study characteristics

We included 11 cohort studies (Table 1): three specifically addressed the “chapeau de gendarme” semiology (one with a temporal lobe origin), five focused on frontal lobe epilepsy (including cases involving the SSMA, orbito‐frontal regions, and FCD in the superior frontal sulcus), two on operculo‐insular epilepsy, and one on FCD without a specific localization. Additionally, we included 16 single case reports: six focused specifically on the “chapeau de gendarme” sign and 10 involved MRI‐negative patients, with invasive exploration performed in eight cases (Table 2). The studies spanned 13 countries, covering a study period from 1988 to 2021. Cohort studies had a mean sample size of 21 patients (SD 16, range 2–60), with an average of four patients per study presenting with the “chapeau de gendarme” semiology (SD 4, range 1–12).

**TABLE 1:** Characteristics of studies including at least two patients presenting with “chapeau de gendarme”/pouting.

First author, year	Study origin	Study period	Number of patients in total	Number of patients with “chapeau de gendarme”	Description/definition of “chapeau de gendarme”	Focus of the study
Sitthinamsuwan,^11^ 2016	Japan	NA	22	9 (12)^a^	Bilateral tonic facial contraction; ictal pouting	Asymmetric tonic seizures/SSMA
Souirti,^1^ 2014	France	1992–2011	36	11	Ictal pouting, with the mouth turned down like a “chapeau de gendarme”/symmetrical and sustained (>5 s) lowering of labial commissures with chin contraction	“chapeau de gendarme”
Lu,^2^ 2021	China	2017–2019	10	10	Downturned appearance of the mouth, held symmetrically, produced by bilateral lip movement, commonly accompanied by chin contraction	“chapeau de gendarme”
Yu,^5^ 2018	China	2011–2015	13	7	“chapeau de gendarme” or pouting	MEG in operculo‐insular epilepsy
Zhao,^6^ 2021	China	2015–2018	27	4	Pouting	Orbito‐frontal epilepsy
Xu,^21^ 2021	China	2016–2017	3	3	Ictal pouting	FCD‐associated epilepsy
Cebeci,^3^ 2019	Turkey	NA	60	3	Ictal pouting, “chapeau de gendarme” sign, defined by a downturned mouth with symmetrical contraction of the lips and chin, resembling a gendarme's hat	Pediatric “chapeau de gendarme” of temporal origin
Wang,^7^ 2019	China	2015–2018	22	2	Pouting	Operculo‐insular epilepsy
Zhang,^8^ 2019	China	2015–2019	17	2	Ictal pouting	FCD in superior frontal sulcus
Chibane,^9^ 2017	Canada	1988–2014	16	2	Ictal pouting	Orbito‐frontal epilepsy
van Dalen,^13^ 2024	UK	2010–2021	50	2	“chapeau de gendarme”	Frontal lobe epilepsy

^a^ The “chapeau de gendarme” sign was described in 12 patients, though semiology could be linked to a specific brain region in only 9 of these cases.

**TABLE 2:** Characteristics of studies describing a single patient with “chapeau de gendarme” or ictal pouting, including case reports.

First author, year	Study origin	Study period	Number of patients in total	Description/definition of “chapeau de gendarme”
Wiwchar,^15^ 2019	Canada	NA	1	“chapeau de gendarme” sign or ictal pouting
Jayapaul,^22^ 2021	India	NA	1	Stereotyped, prolonged inverted ictal pouting
Lentoiu,^17^ 2022	Romania	2012–2021	2	Pouting: corners of the mouth pointing downwards
Wang,^23^ 2018	China	2015–2016	14	Pouting
Chou,^24^ 2020	Taiwan	NA	18	Pouting
Ciurea,^10^ 2015	Romania	NA	1	Grimacing (“chapeau de gendarme”)
Tan,^25^ 2016	USA	NA	1	Ictal pouting, chapeau de gendarme: downward turning of the mouth (“as if she was really sad and about to cry”)
Hayakawa,^12^ 2018	Japan	NA	1	Ictal pouting, chapeau de gendarme, Kabuki visage: jaw tightening, depressed angle of the mouth, resembles the face of an actor in Kabuki
Koc,^16^ 2017	Turkey	NA	1	Tearful appearance, inverted smile, mouth turned down with symmetric pucking of lips, lowering of the labial corners, and contraction of the chin at the end
Marques,^26^ 2023	Canada	NA	1	Ictal pouting, chapeau de gendarme
Ruesch,^4^ 2021	Switzerland	NA	1	Ictal pouting
Peltola,^27^ 2020	France, Finland	2010–2017	11	Ictal pouting
de Campos,^28^ 2019	Portugal	NA	1	Change in facial expression suggesting disgust (with sustained downward movement of labial commissures, i.e., pouting)
Li,^29^ 2021	China	NA	1	Ictal pouting
Beltrán‐Corbellini,^30^ 2024	Spain	NA	1	Bilateral oral commissure down‐slanting (“chapeau de gendarme” sign)
Wang,^31^ 2020	China	2015–2018	22	Pouting

Six studies from the same site were identified: two by the same author^7^, ^32^ and four with overlapping co‐authors.^5^, ^6^, ^8^, ^33^ A detailed review of individual patient characteristics confirmed no overlap in patients; thus, all six studies were included. However, two additional studies by the same author involved overlapping patient cohorts.^2^, ^34^ The study with the smaller sample size was excluded^34^ as its patients were already represented in the other study.^2^

**FIGURE 2:**
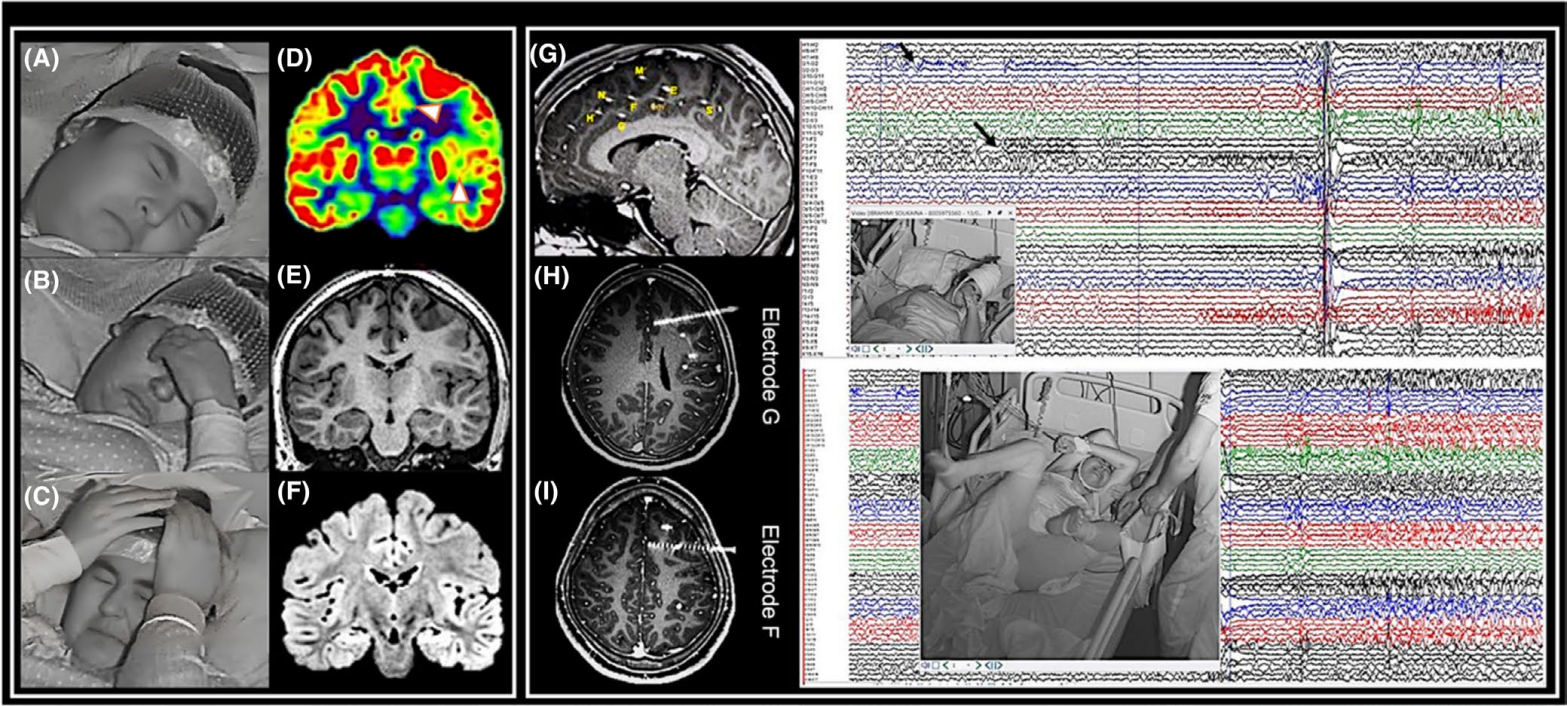
“Chapeau de gendarme” in a 16‐year‐old girl with sleep‐related hypermotor epilepsy. Left panel: Scalp EEG recording shows early occurrence of the “chapeau de gendarme” sign (5 s after the first clinical change), accompanied by forced eye closure and facial grimacing, preceding the hypermotor phase. Photos (A, B, C) illustrate the sustained component of the sign (7‐s duration) and the sequential chronology of mouth and chin contraction. FDG‐PET reveals hypometabolic areas involving the anterior/middle cingulate gyrus, the central operculum, and the insula on the left side, with no corresponding abnormalities on MRI. Right panel: SEEG recording from the same patient. The implantation scheme shows the electrode placement (insulo‐opercular electrodes not represented). Electrodes G and F are located in the cingulate gyrus (at the junction of the anterior and middle cingulate parts). The discharge originates in the cingulate gyrus (arrows), coinciding with the first clinical symptom: A non‐painful sensation in the eyes, followed by hand‐to‐eye movement, forced eye closure, and the “chapeau de gendarme” sign. Propagation to the opercular areas (Op) occurs secondarily. During the hypermotor phase, the “chapeau de gendarme” persists and correlates with extensive mesial frontal involvement. The patient achieved seizure freedom following a resection limited to the cingulate gyrus around electrodes G and F. Histopathology confirmed focal cortical dysplasia type IIA (FCD2A).

“Chapeau de gendarme” was originally defined as a “symmetrical and sustained (>5 s) lowering of labial commissures with chin contraction, mimicking an expression of fear, disgust, or menace.”^1^ However, semiologic descriptions varied across studies, with terms including “pouting,” “bilateral tonic facial contraction,” “symmetrical down‐turned mouth,” “grimacing,” “inverted smile with a tearful expression,” “mouth turning down with symmetric puckering,” and “labial corners lowered with chin contraction” (Figure 2). A video analysis study suggested that the “chapeau de gendarme” sign has two components^2^: a “prodromal component,” marked by vertical constriction of the lips and chin resembling forced mouth closure, and a “major component,” with symmetrical downward contraction of the mouth corners. In some cases, the “prodromal component” was nearly imperceptible, likely due to preictal/ictal behaviors or merging with the major component.

Importantly, the time of occurrence (primary vs. secondary), the duration of the sign (brief or prolonged), and the associated features (facial expression and motor behavior) must be carefully considered to differentiate the various types of mouth contraction and the neural networks underlying this manifestation.

### 3.3 Patient characteristics

**FIGURE 3:**
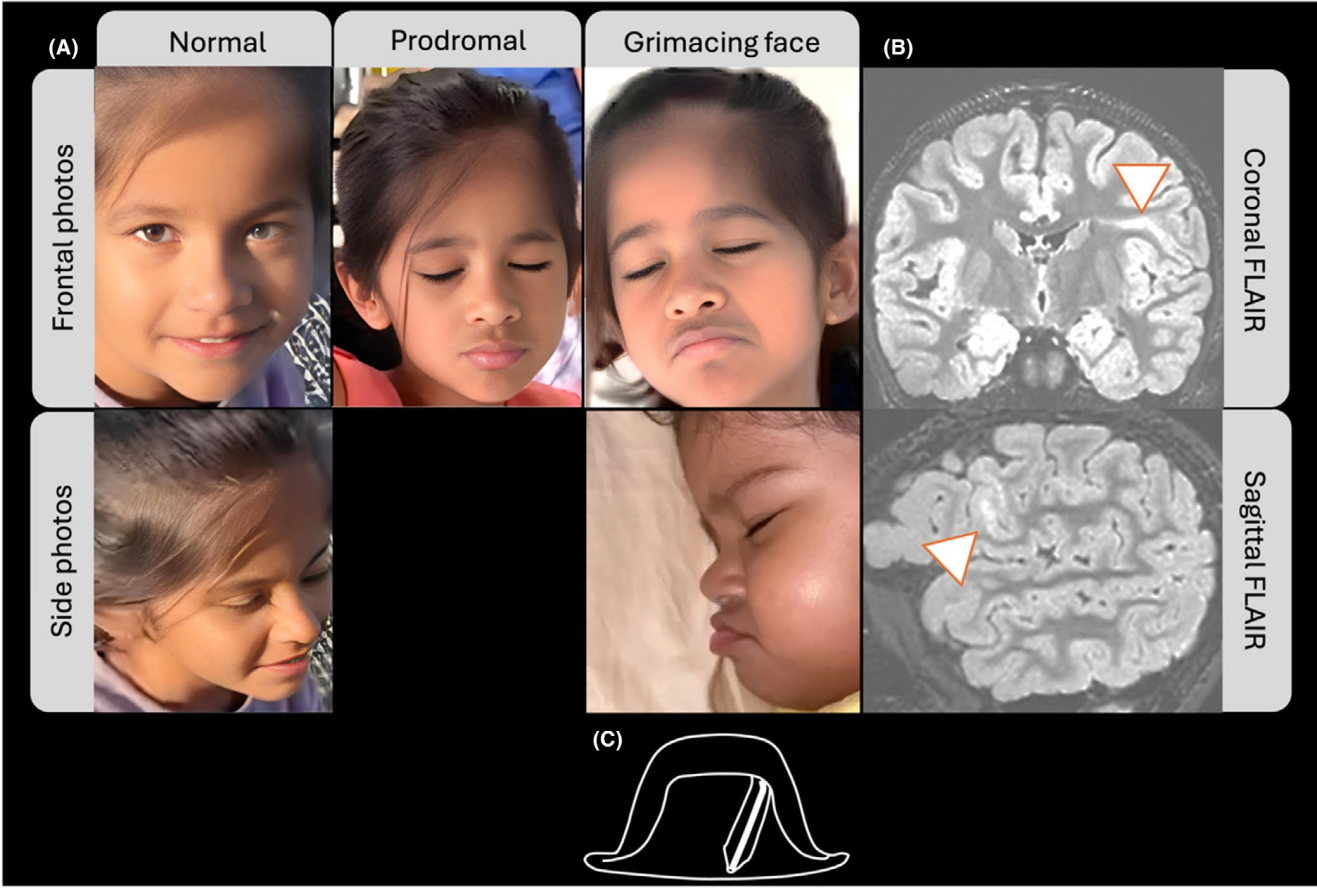
The “chapeau de gendarme” sign in a young girl, illustrated at different stages of her epilepsy and the development of this semiology. (A) Evolution of ictal semiology: Starting with a neutral facial expression, progressing to the prodromal stage characterized by vertical lip contraction, and culminating in the ictal phase with a downward‐turned mouth and symmetrical contraction of the lips and chin, resembling the “chapeau de gendarme” sign. (B) MRI images from the same patient demonstrating a left‐sided focal cortical dysplasia. Key features include cortical thickening (white arrowhead on the sagittal image), the transmantle sign (white arrowhead on the coronal image) and FLAIR hyperintensity of the subcortical white matter. (C) Illustration of a gendarme hat from the French Revolution, emphasizing its resemblance to the observed facial expression. FLAIR, fluid‐attenuated inversion recovery.

Of 71 patients in our cohort (Tables 3 and 4), 52% were male and 41% female; in 7% data regarding the sex was not provided. Adults made up the majority of cases (62%), with 37% pediatric cases (1% unknown; Figure 3). The mean age at epilepsy onset was 8.8 years (SD 6, range 0–28). MRI was negative in 49% of cases and positive in 34%; results were not reported in 17% of cases. Among MRI‐positive cases, FCD was the predominant radiological diagnosis, accounting for 96% of cases, with a single tumor case representing the remaining 4%. Fluorodeoxyglucose positron emission tomography (FDG‐PET) was performed in 62% of cases and consistently indicated hypometabolism, including in MRI‐negative patients, with 95% showing hypometabolism and only 5% yielding “non‐localizing” results.

**TABLE 3:** Patient characteristics in studies that included at least two patients presenting with “chapeau de gendarme.”

First author, year	Adults (A) children (P)	Mean age at epilepsy onset (SD, range) in years	Mean age at surgery (SD, range) in years	% MRI positive	% SEEG	% operated	% class IA outcome ≥1 year	Confidence in the EZ (% very high/high/moderate/low)
Sitthinamsuwan,^11^ 2016	8 (A) 4 (P)	NA	22 (9, 7–32)	NA	100	100	56	56/23/0/23
Souirti,^1^ 2014	9 (A) 2 (P)	8 (4, 3–17)	30 (13, 16–48)	45	82	100	100	100/0/0/0
Lu,^2^ 2021	7 (A) 3 (P)	NA	21 (8, 8–34)	40	100	100	70	70/30/0/0
Yu,^5^ 2018	5 (A) 2 (P)	11 (7, 4–24)	24 (13, 14–50)	0	100	100	86	86/0/0/14
Zhao,^6^ 2021	1 (A) 3 (P)	6 (5, .3–13)	18 (11, 6–33)	100	100	100	100	100/0/0/0
Xu,^21^ 2021	2 (A) 1 (P)	4 (3, 1–6)	16 (6, 9–21)	67	100	100	0	0/100/0/0
Cebeci,^3^ 2019	3 (P)	7 (6, 2–14)	13 (4, 9–16)	67	0	0	–	0/66/0/33
Wang,^7^ 2019	1 (A) 1 (P)	7 (5, 3–10)	15 (14, 4–25)	0	100	100	100	100/0/0/0
Zhang,^8^ 2019	1 (A) 1 (P)	12 (10, 5–19)	22 (8, 16–28)	50	100	100	100	100/0/0/0
Chibane,^9^ 2017	2 (A)	13 (6, 9–17)	33 (8, 27–38)	0	100	100	100	100/0/0/0
van Dalen,^13^ 2024	2 (P)	NA	NA	NA	NA	100	100	100/0/0/0

**TABLE 4:** Patient characteristics in articles describing a single patient with “chapeau de gendarme” or ictal pouting, including case reports.

First author, year	Adults (A) children (P)	Age at epilepsy onset in years	Age at surgery in years	MRI positive	SEEG	Operated	Class IA outcome ≥1 year	Confidence in the EZ
Wiwchar,^15^ 2019	P	6	17	No	Yes	Yes	Yes	Very high
Jayapaul,^22^ 2021	A	19	25	No	Yes	Yes	No	High
Lentoiu,^17^ 2022	A	NA	39	No	Yes	Yes	Yes	Very high
Wang,^23^ 2018	NA	NA	NA	No	No	Yes	Yes	Very high
Chou,^24^ 2020	A	NA	39	No	Yes	Yes	No	High
Ciurea,^10^ 2015	A	3	33	No	Yes	Yes	Yes	Very high
Tan,^25^ 2016	A	1.5	28	Yes	Yes	Yes	No	High
Hayakawa,^12^ 2018	P	15	16	Yes	No	Yes	No	High
Koc,^16^ 2017	A	5	24	Yes	No	Yes	No	High
Marques,^26^ 2023	A	NA	65	Yes	Yes	Yes	No	High
Ruesch,^4^ 2021	P	1.2	–	Yes	No	No	–	High
Peltola,^35^ 2020	P	.8	7	No	Yes	Yes	Yes	Very high
de Campos,^28^ 2019	A	17	–	Yes	No	No	–	High
Li,^29^ 2021	A	7	27	No	Yes	Yes	Yes	Very high
Beltrán‐Corbellini,^30^ 2024	A	10	50	No	Yes	No	No	High
Wang,^31^ 2020	A	28	NA	NA	NA	Yes	Yes	Very high

**FIGURE 4:**
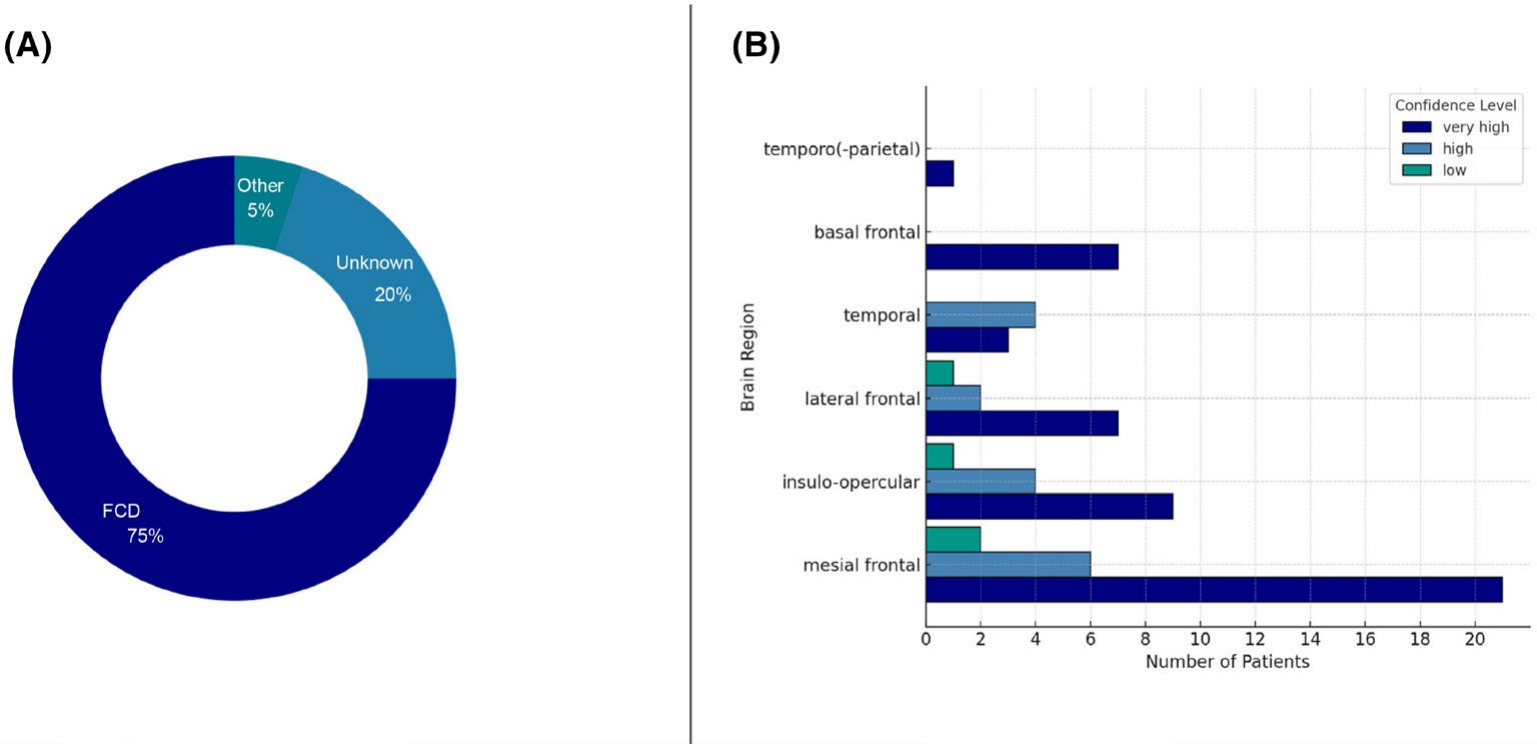
(A) Histopathological diagnoses of resected tissue from patients who presented the “chapeau de gendarme” sign during presurgical evaluation and later underwent resective epilepsy surgery. (B) Confidence levels in identifying the epileptic zone (EZ) within different brain regions based on neuroimaging, invasive EEG, and postsurgical seizure outcomes. The EZ was most commonly located in the mesial frontal region (21 with very high confidence, 6 high, 2 low), followed by the insulo‐opercular region (9 very high, 4 high, 1 low), lateral frontal region (7 very high, 2 high, 1 low), temporal region (3 very high, 4 high), and basal frontal region (7 very high). In a single case,^17^ the EZ was localized to the posterior part of the middle temporal gyrus and the inferior parietal region and is therefore listed separately.

iEEG was performed in 82% of patients, with one patient subsequently undergoing thermocoagulation. Resective epilepsy surgery was performed in 92% of patients, with a mean age at surgery of 24 years (SD 12, range 4–65). Surgery was more frequently performed on the right side (45%) compared to the left side (36%; 15% unknown). Following resective surgery, 77% of patients remained seizure‐free (Engel class Ia) over a postsurgical follow‐up duration of at least 1 year. FCD was the most common histopathological finding, identified in 75% of cases: 59% were FCD type 2, 11% FCD type 1, and 23% FCD not further specified. The remaining 7% included tumors or vascular malformations (Figure 4A). Other pathologies included glioma, glioblastoma, cavernous angioma, and arteriovenous malformation.

### 3.4 Assessment of bias

The overall risk of bias was high, primarily due to the descriptive nature of the studies (Table 5). This design, necessary to link semiology to clinical characteristics, introduced a high risk of selection bias (63% high vs. 38% low). Assessment bias was rated as high or unclear in most cases (44% unclear, 44% high, 13% low).

**TABLE 5:** Assessment of Bias.

First author, year	Selection bias	Assessment bias
Sitthinamsuwan,^11^ 2016	Low	High
Souirti,^1^ 2014	Low	High
Lu,^2^ 2021	Low	High
Yu,^5^ 2018	High	Low
Zhao,^6^ 2021	Low	Low
Xu,^21^ 2021	Low	Unclear
Cebeci,^3^ 2019	High	High
Wang,^7^ 2019	High	Unclear
Zhang,^8^ 2019	High	Unclear
Chibane,^9^ 2017	High	Unclear
van Dalen,^13^ 2024	High	High
Wang,^23^ 2018	Low	Unclear
Lentoiu,^17^ 2022	High	High
Chou,^24^ 2020	High	Unclear
Peltola,^35^ 2020	High	High
Wang,^31^ 2020	High	Unclear

*Note*: Only cohort studies were evaluated. Selection bias was assessed based on whether studies enrolled a consecutive or random patient sample, clearly defined the sampling method, used a case–control design, and avoided inappropriate exclusions. Assessment bias was evaluated by determining if the semiology assessment was blinded to other data.

### 3.5 Anatomo‐clinical correlations and confidence in epileptic zone localization

In 43% of cases, the EZ was localized to the mesial frontal region, with additional localizations in the insulo‐opercular (21%), lateral frontal (15%), basal frontal (10%), temporal (10%), and temporo‐parietal (1%) regions. Seizure onset was more frequently associated with the mesial and lateral frontal regions (38% and 50%, respectively), while basal frontal and insulo‐opercular regions were more commonly involved during propagation (29% and 21%, respectively). However, distinguishing whether the “chapeau de gendarme” semiology reflected seizure onset or propagation remained challenging, as a large proportion of cases (30%–72%, depending on the region) lacked precise temporal information (Table 6).

**TABLE 6:** Anatomo‐clinical correlations.

Brain region	% reported	More at onset or during propagation (limited data)	Overall grade level (high, moderate, low) that CDG is associated with the brain region
Mesial frontal	43	Onset (38% at Onset, 17% at Propagation, 45% unknown)	High
Basal frontal	10	Propagation (14% at Onset, 29% at Propagation, 57% unknown)	Low (limited data)
Lateral frontal	15	Onset (50% at Onset, 20% at Propagation, 30% unknown)	Moderate/Low
Insulo‐opercular	21	Propagation (7% at Onset, 21% at Propagation, 72% unknown)	Moderate
Temporal	10	Onset (29% at Onset, 14% at Propagation, 57% unknown)	Low (limited data)
Temporo(−parietal)	1	Propagation	Low (limited data)

*Note*: Determining whether the “chapeau de gendarme” semiology indicated seizure onset or propagation was challenging due to limited data.

In 72% of patients, localization confidence for the EZ was rated as very high (Figure 4B), predominantly in the mesial frontal region (41% mesial frontal vs. 14% basal frontal, 14% lateral frontal, 6% frontal without sublobar specification, 17% insulo‐opercular, 6% temporal, and 2% temporo‐parietal). In addition, 22% of patients had a high confidence in their EZ localization, again most commonly in the mesial frontal region (38% mesial frontal, 12% lateral frontal, 25% insulo‐opercular, and 25% temporal). Even in cases with low confidence, the mesial frontal region was the predominant localization (50% mesial frontal, 25% lateral frontal, 25% insulo‐opercular).

**FIGURE 5:**
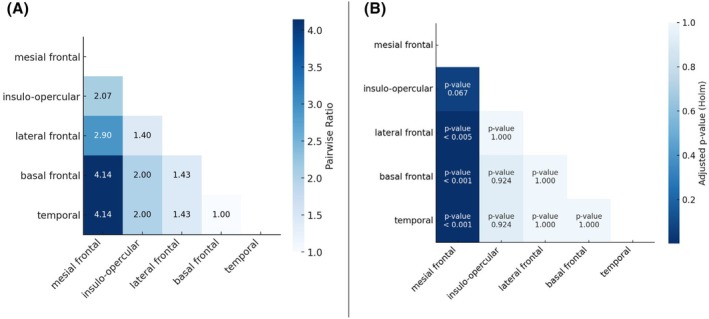
(A) Pairwise ratios of brain region occurrences, calculated as the ratio of the region on the *x*‐axis to the region on the *y*‐axis. Darker shades represent higher ratios. (B) Pairwise differences in occurrence ratios across brain regions, adjusted for multiple comparisons using the Holm correction. Darker shades indicate smaller *p*‐values (more significant differences), while lighter shades indicate less/no significant differences.

The pairwise ratios and differences in occurrence ratios across brain regions, adjusted for multiple comparisons using the Holm correction, are shown in Figure 5. The single case of temporo‐parietal EZ localization was excluded to avoid potential bias in the pairwise ratio analysis. Among all regions, the mesial frontal region had the highest occurrence rate, with statistically significant differences compared to all other regions. Specifically, its occurrence rate was 4.14 times higher than in the basal frontal and temporal region (*p* < .001), 2.90 times higher than the lateral frontal region (*p* = .0049), and 2.07 times higher than the insulo‐opercular region (*p* = .07). No significant differences were detected among the other regions.

### 3.6 Overall summary of evidence

There is strong evidence associating the “chapeau de gendarme” semiology with frontal (Table 6), particularly mesial frontal, EZ localization, with moderate evidence suggesting a potential origin in the insulo‐opercular cortex.

## 4 DISCUSSION

Our systematic review provides strong evidence supporting the “chapeau de gendarme” sign as a localizing feature in focal epilepsy, primarily linked to the mesial frontal regions, with moderate evidence suggesting involvement of the insulo‐opercular regions. These findings highlight the potential value of the “chapeau de gendarme” sign in identifying the EZ, particularly in cases where structural imaging is inconclusive.

### 4.1 Does the “chapeau de gendarme” sign indicate the seizure origin?

The first study^1^ (*N* = 11) describing the “chapeau de gendarme” as a localizing feature identified the ACC as the primary region of generation in all but one case, where the sign originated in the insulo‐opercular cortex with secondary spread to the ACC. The sign was associated with negative emotions such as disgust, distress, displeasure, disappointment, disapproval, disagreement, and doubt, interpreted as a defensive or negative behavioral response to stimuli with affective or cognitive components. Stronger emotional expressions, such as fear or menace, with open eyes, hypermotor, and autonomic features, were linked to the rostroventral “affective” ACC.^1^ Milder expressions, such as discontent, disappointment, disagreement, or doubt, with closed eyes and without hypermotor features, were linked to the dorsal “cognitive” ACC.^1^ Notably, all these mimics can also be observed in healthy subjects, where they serve as a non‐verbal modality of social interaction to convey various messages. This suggests that they are part of an integrated behavior involving emotional and affective expression, rather than an unnatural motor behavior as interpreted by some authors.^36^, ^37^ We propose that the primary neural substrate for ictal pouting is cortical, centered in the anterior cingulate cortex, with strong modulatory input from the insula and likely downstream involvement of subcortical emotional processing hubs. It is best understood as a behavioral–emotional expression rather than a purely motor output driven by stereotyped pattern generators.

These findings align with ACC‐originating semiologies observed in spontaneous^38^, ^39^, ^40^ or elicited seizures,^24^, ^30^ which often involve hyperkinetic behaviors, mood changes such as fear or menace, aggressive verbalizations or actions, and oral movements while retaining interaction. The “chapeau de gendarme” sign likely reflects pathological activation of prefrontal circuits involved in goal‐directed behaviors or emotional responses.^2^ However, the sign has been also observed in tonic seizures arising from the supplementary sensorimotor area (SSMA; *N* = 9),^11^ where activation triggers proximal and truncal muscle contractions, suggesting this as a common but non‐specific feature of SSMA epilepsy.^11^ Additional adult^2^, ^10^, ^16^, ^29^, ^41^, ^42^ and pediatric^13^ case studies further support the mesial frontal origin of the “chapeau de gendarme” sign.

Moderate evidence also links the “chapeau de gendarme” sign to the insulo‐opercular regions, supported by strong connections between the antero‐mid cingulate and the antero‐mid limbic insular cortex,^1^, ^2^ facilitating complex viscerosomatic and higher‐order emotional and cognitive processing. The anterior insula, known for its role in processing social emotions such as disgust and fear,^22^, ^25^ has been implicated in generating this sign. The involvement of the insular cortex was first raised in the initial study describing the sign,^1^ which emphasized the importance of a network including the insula.^1^ A study on insulo‐opercular epilepsy (*N* = 7)^5^ and several adult^35^, ^43^ and pediatric^2^, ^4^, ^7^, ^15^, ^27^ case studies further support this association. Notably, both the insular and opercular regions are commonly involved in the EZ,^7^ rendering differentiation between seizure onset in these areas challenging. SEEG plays a critical role in clarifying the roles of the insulo‐opercular regions and their interaction with interconnected brain regions.^26^, ^44^ To optimize EZ localization and surgical outcomes, the insula should be systematically included in SEEG explorations for patients presenting with the “chapeau de gendarme” sign.

Additional frontal lobe regions, including the basal frontal,^1^, ^45^ lateral frontal,^1^, ^3^, ^8^, ^34^ and other areas^21^ have also been associated with the “chapeau de gendarme” sign in both adult^1^, ^9^, ^34^, ^45^ and pediatric^1^, ^3^, ^45^ case studies. The propagation of ictal discharges from the lateral to medial frontal cortex^2^, ^33^, ^36^ may involve prefrontal‐cingulate inhibition via feedforward projections from the supragranular layers of the dorsolateral prefrontal cortex to the deeper layers of the anterior‐middle cingulate cortex. This pathway could allow discharges originating in the lateral prefrontal cortex, with its more complex laminar structure, to activate the emotional insulo‐cingulate network, resulting in exaggerated behaviors often accompanied by the “chapeau de gendarme” sign.^3^ The orbito‐frontal cortex, with its extensive reciprocal connections to the frontal lobe, temporal lobe, and limbic structures,^45^ may also contribute to this sign through its cognitive functions, including reward processing, risk control, and learning. Recognizing that these regions may form part of a broader network underlying the “chapeau de gendarme” sign is crucial for interpreting auxiliary neuroimaging findings, such as PET, and for planning SEEG investigations, particularly in MRI‐negative cases.

Some adult^2^, ^7^, ^17^ and pediatric^3^, ^12^ case studies^31^ suggest that the temporal region may also contribute to the “chapeau de gendarme” sign, either through direct involvement of the insulo‐opercular regions in the EZ^3^, ^7^ or through early propagation.^2^ Robust amygdaloid projections to the limbic cortex, including the ACC, anterior midcingulate cortex, and agranular insula, may facilitate the spread of epileptic discharges from the anterior temporal cortex into the insulo‐cingulate network through the anterior medial temporal regions, particularly the amygdala and peri‐amygdaloid areas.^2^ The role of the insulo‐opercular cortex in temporal lobe seizures with oro‐alimentary automatisms has also been demonstrated.^46^ However, the rarity of “chapeau de gendarme” sign in seizures confined to insulo‐opercular structures without significant temporal lobe involvement highlights the importance of specific network dynamics in generating such complex ictal behaviors.^47^

This distribution across several brain lobes suggests that the semiology may result from a broader network involving multiple interconnected areas. A major challenge remains in determining whether the “chapeau de gendarme” sign indicates seizure onset or propagation. The currently available data do not allow for clear differentiation, largely due to insufficient temporal resolution in many reports, with 30%–72% of cases lacking information on the timing of the semiology relative to seizure onset. Nonetheless, in cases where timing was reported, the sign most often appeared early in the seizure, particularly in mesial and lateral frontal epilepsies, suggesting that it may reflect early engagement of frontal symptomatogenic zones rather than late propagation.

Finally, while several studies have supported the utility of the “chapeau de gendarme” sign in localizing the EZ, no apparent lateralizing value has been attributed to this semiology.^1^

### 4.2 Does the “chapeau de gendarme” sign indicate the epilepsy substrate?

In our systematic review, FCD was the most common radiological diagnosis, identified in all but one MRI‐positive cases, and the most frequent histopathological finding among patients who underwent resective surgery, present in 75% of cases, including FCD type 2 in 59% of cases where specified. Other pathologies, such as tumors and vascular malformations, were reported less frequently in relation to the “chapeau de gendarme” sign. However, this pattern does not imply a specific link between the “chapeau de gendarme” sign and FCD, particularly FCD type 2. The co‐occurrence may be incidental, given that FCD type 2 is a common finding in patients undergoing invasive recordings, especially in extratemporal epilepsy with negative MRI, where other etiologies such as tumors are less prevalent. Rather, the sign appears to be associated with focal networks originating from specific brain regions, with FCD type 2 serving as a common substrate in focal epilepsy cases. Conversely, ictal semiology highly specific to brain localization has been reported as a characteristic clinical phenotype in FCD type 2 studies.^48^, ^49^ Moreover, the notably high proportion of FCD type 2 associated with the “chapeau de gendarme” sign suggests that this semiology could serve as a potential clinical marker for FCD type 2, particularly in MRI‐negative cases.

Nearly half (49%) of the focal epilepsy cases in our review were MRI‐negative, highlighting the importance of localizing signs such as the “chapeau de gendarme” in focal lesional epilepsy in cases where structural MRI fails to detect the epileptogenic lesion.^50^, ^51^, ^52^ FDG‐PET proved particularly valuable in these MRI‐negative cases, especially when scalp EEG findings were non‐lateralizing or non‐localizing. By consistently identifying areas of hypometabolism, FDG‐PET facilitated further investigation with SEEG to delineate the EZ, further supporting its role as an auxiliary tool in the presurgical evaluation of focal epilepsy.

### 4.3 Clinical value of the “chapeau de gendarme” sign

The “chapeau de gendarme” sign is a distinctive feature of focal epilepsy and holds significant value in the presurgical evaluation of patients with refractory seizures across all age groups.^4^, ^13^ This semiology should prompt consideration of a mesial frontal origin and the possibility of seizure propagation from regions closely connected to mesial frontal structures.^1^ Screening for FCD is particularly relevant, as FCDs are the most common substrate in patients with drug‐resistant focal lesional epilepsy, particularly in young children.^32^, ^53^, ^54^, ^55^, ^56^ Recognizing episodic “pouting” as a potential indicator of focal epilepsy is crucial and warrants further investigation through EEG and MRI.^4^ FCD type II, often located in extratemporal regions such as the frontal lobe,^57^ is especially relevant in these cases. Surgical intervention targeting FCD type II has shown high rates of postsurgical seizure freedom,^53^ underscoring the importance of identifying this semiology to guide treatment strategies.

While frontal lobe networks underlying specific semiologies have been identified,^36^ pinpointing sublobar origins of frontal lobe seizures based on semiology alone remains challenging due to extensive interregional connectivity and rapid seizure propagation. Isolated signs or symptoms have limited value for localizing the EZ, and electrical activity at seizure onset alone cannot fully explain the emergence of specific semiologies.^58^ The “chapeau de gendarme” sign should therefore be evaluated as part of a comprehensive presurgical assessment, integrating electrophysiological and neuroimaging data to improve accuracy in localizing the EZ and optimizing surgical outcomes.

### 4.4 Limitations

The limitations of our systematic review primarily arise from biases in the included studies and variability in the semiologic descriptions of the “chapeau de gendarme” sign, as comprehensive photo or video documentation was lacking in most cases. Notably, only three studies^1^, ^2^, ^3^ specifically addressed this semiology, suggesting it may often be overlooked in other cohorts or clinical contexts. The descriptive nature of these studies, the insufficient detail on seizure onset and propagation further limits the strength of associations between this semiology and specific localizations or pathologies.

Despite these limitations, the “chapeau de gendarme” sign remains a valuable feature. Our findings emphasize the need for standardized, homogeneous descriptions, consistent reporting, and detailed documentation to improve its recognition. Regarding the term “chapeau de gendarme,” it should retain its seminal description, which includes the sustained character of the mouth contraction (lasting long enough to allow a photo to be taken), bilateral symmetric lowering of the labial commissures, and contraction of the chin – mimicking an expression of fear, disgust, or menace, as outlined in the ILAE semiologic glossary.^59^

## 5 CONCLUSION

The “chapeau de gendarme” sign is a distinctive semiology associated with focal epilepsy, primarily involving the mesial frontal regions, with additional possible involvement of insulo‐opercular and temporal networks. Its identification can aid in localizing the EZ, particularly in cases of suspected FCD, one of the most common substrates of refractory focal epilepsy, especially in children. While variability in descriptions and interpretations complicates its consistent recognition, the presence of this sign warrants detailed investigation using EEG, MRI, and FDG‐PET, especially in MRI‐negative cases. Integrating associated ictal symptomatology with electrophysiological and neuroimaging findings can enhance diagnostic accuracy and inform surgical strategies. The clinical relevance of the “chapeau de gendarme” sign underscores the need for consistent reporting and further research to refine its diagnostic utility.

## FUNDING INFORMATION

This study was supported by project grants from the Swiss National Science Foundation (SNSF: 208184) to GR and the Anna Mueller Grocholski and Theodor und Ida Herzog‐Egli‐Stiftung to AGG.

## CONFLICT OF INTEREST STATEMENT

None of the authors has any conflict of interest to disclose. We confirm that we have read the Journal's position on issues involved in ethical publication and affirm that this report is consistent with those guidelines.

**Test yourself**

1. What characterizes the chapeau de gendarme facial expression?
  - Unilateral facial twitching and grimacing
  - Bilateral downward turning of the mouth and chin contraction
  - Eyelid fluttering and forced eye deviation
  - Jaw clenching with upper lip retraction
2. What is the chapeau de gendarme sign most commonly associated with?
  - Occipital lobe epilepsy
  - Temporal lobe epilepsy
  - Mesial frontal lobe epilepsy
  - Parietal lobe epilepsy
3. Which histopathological finding is most frequently linked to the chapeau de gendarme sign?
  - Hippocampal sclerosis
  - Dysembryoplastic neuroepithelial tumor (DNET)
  - Focal cortical dysplasia type II
  - Ganglioglioma
4. What is the primary clinical value of identifying the chapeau de gendarme sign in seizure semiology?
  - It confirms generalized epilepsy
  - It predicts pharmacoresistance
  - It aids in localizing the epileptogenic zone
  - It identifies seizures with autonomic features

*Answers may be found in the* *supporting information*.

## Supporting information


Tables S1–S4:

Appendix S1

## Data Availability

Data sharing not applicable to this article as no datasets were generated or analysed during the current study.
