# Supplementary material for: The “chapeau de gendarme” sign in focal epilepsy: A systematic review
Source: Epileptic Disord. 2025 May 27;27(5):867–82. doi: 10.1002/epd2.70048 (PMC12574494; doi:10.1002/epd2.70048)
Supplement: Supplementary file 1 — Tables S1–S4: [file EPD2-27-867-s001.docx]

**SUPPLEMENTARY TABLES**

**Supplementary Table 1:** Search Strategy for PubMed.

| Operator | Search |
| --- | --- |
|  | "Chapeau de gendarme"[all] OR "pout*"[all] OR “ictal pout*”[all] OR “kabuki visage”[all] OR "facial"[tw] OR "grima*"[tw] OR "Emotions"[Mesh] |
| AND | "Epilepsies, Partial"[Mesh] OR "focal epilep*"[tw] OR "Seizures"[Mesh] OR "seizure*"[tw] |
| AND | ("Neurosurgical Procedures"[Mesh] OR "Seizures/surgery"[MeSH] OR "epilepsy surgery"[tw]) OR ("Electroencephalography"[Mesh] OR “electroencephalogra*”[tw] OR “EEG” [tw]) |
| AND | "Electrodes, Implanted" [MAJR] OR "Intracranial electroencephalogra*"[tw] OR "Intracranial EEG"[tw] OR “Stereoelectroencephalogra*”[tw] OR “SEEG” [tw] OR "seizure onset"[tw] OR "epileptogenic zone"[tw] OR “semiology” [tw] |
| NOT | "Epilepsy, Generalized"[Mesh] OR “generalized epilep*”[tw] |
| NOT | “psychogenic nonepileptic seizures” [tw] OR “PNES” [tw] |
| NOT | animals[mh] NOT humans[mh] |

**Supplementary Table 2:** Search Strategy for EMBASE.

| Operator | Search |
| --- | --- |
|  | 'chapeau de gendarme: ti, ab, kw, de' OR 'pout*: ti, ab, kw, de' OR 'ictal pout*: ti, ab, kw, de' OR 'kabuki visage: ti, ab, kw, de' OR 'facial' OR ‘grima*’ OR 'emotion'/exp OR 'emotion' |
| AND | 'focal epilepsy'/exp OR 'focal epilep*' OR 'focal seizures'/exp OR 'seizure*' |
| AND | ('epilepsy surgery'/exp OR 'epilepsy surgery') OR ('electroencephalogram'/exp OR ‘electroencephalogra*’ OR 'EEG') |
| AND | 'electrodes, implanted'/exp/mj OR 'intracranial electroencephalogra*' OR 'intracranial EEG' OR 'stereoelectroencephalography'/exp OR 'stereoelectroencephalogra*' OR 'SEEG' OR 'seizure onset' OR 'epileptogenic zone'/exp OR 'epileptogenic zone' OR 'semiology’ |
| NOT | 'generalized epilepsy'/exp OR 'generalized epilepsy' |
| NOT | 'nonepileptic seizure'/exp OR 'nonepileptic seizure' OR 'pnes' |
| NOT | ('animal'/exp OR 'animal') NOT ('human'/exp OR 'human') |

**Supplementary Table 3:** Search Strategy for SCOPUS.

| Operator | Search |
| --- | --- |
|  | ALL ({Chapeau de gendarme} OR pout* OR “ictal pout*” OR {kabuki visage} OR facial OR grima* OR emotion) |
| AND | TITLE-ABS-KEY (“focal epilepsy” OR seizure) |
| AND | TITLE-ABS-KEY (“epilepsy surgery” OR electroencephalogra* OR EEG) |
| AND | TITLE-ABS-KEY (Intracranial electroencephalogra* OR Intracranial EEG OR Stereoelectroencephalogra* OR SEEG OR “seizure onset” OR “epileptogenic zone” OR semiology) |
| AND NOT | TITLE-ABS-KEY (“generalized epilepsy”) |
| AND NOT | TITLE-ABS-KEY (“psychogenic nonepileptic seizures” OR PNES) |

**Supplementary Table 4:** Scoring sheet for full-text screening.

## **THE ‘CHAPEAU DE GENDARME’ SIGN IN FOCAL EPILEPSY:**

## **A SYSTEMATIC REVIEW**

Assessment sheet for full-text screening

Rater: Reviewer 1 Article number (Covidence): __________

Reviewer 2 Scoring date: __________

**Inclusion/ Exclusion criteria**

|  |  | yes | no | NA |
| --- | --- | --- | --- | --- |
| 1. **Type of data** | Data on invasive EEG recordings available? |  |  |  |
|  | Data on epilepsy surgery + postsurgical seizure outcome available? |  |  |  |
|  | Data on brain imaging available? |  |  |  |
| 1. **Data Reporting** | Detailed individual patient information that enables a link between semiology and the localization of the EZ? |  |  |  |
| 1. **Semiological description** | ‘Chapeau de gendarme’ or ‘pouting’ described? |  |  |  |
| 1. **No description of CDG/ pouting/ Grimazing** | Detailed description of semiology or photos providing sufficient evidence to confirm the chapeau de gendarme sign? |  |  |  |
| 1. **Access** | Full text available? |  |  |  |
| 1. **Type of article** | Research article with original data |  |  |  |
|  | Case reports |  |  |  |
|  |  |  |  |  |
|  | **Inclusion criteria fulfilled?** |  |  |  |

🡪 Document reasons for inclusion/ exclusion in Covidence
