# Supplementary material for: The “chapeau de gendarme” sign in focal epilepsy: A systematic review
Source: Epileptic Disord. 2025 May 27;27(5):867–82. doi: 10.1002/epd2.70048 (PMC12574494; doi:10.1002/epd2.70048)
Supplement: Supplementary file 2 — Appendix S1 [file EPD2-27-867-s002.docx]

**TEST YOURSELF**

**Answers:**

1. **B**

2. **C**

3. **C**

4. **C**
